# Prognostic significance of circulating tumor DNA in urothelial carcinoma patients undergoing immune checkpoint inhibitor therapy: a systematic review and meta-analysis

**DOI:** 10.3389/fimmu.2025.1574449

**Published:** 2025-04-29

**Authors:** Qingping Ma, Shufu Hou, Haibo Ma, Jing Gao, Dandan Song

**Affiliations:** ^1^ Shandong Provincial Third Hospital, Cheeloo College of Medicine, Shandong University, Jinan, China; ^2^ Department of Hyperbaric Oxygen, Shandong Provincial Third Hospital, Cheeloo College of Medicine, Shandong University, Jinan, China; ^3^ Department of Gastrointestinal Surgery, Central Hospital Affiliated to Shandong First Medical University, Jinan, China; ^4^ Department of Neurology, Shandong Provincial Third Hospital, Cheeloo College of Medicine, Shandong University, Jinan, China

**Keywords:** urothelial carcinoma (UC), circulating tumor DNA (ctDNA), immune checkpoint inhibitors, overall survival (OS), disease-free survival, progression-free survival

## Abstract

**Background:**

Circulating tumor DNA (ctDNA) has emerged as a novel biomarker with the advantages of being non-invasive and enabling dynamic monitoring, providing significant clinical insights into the prognosis and management of malignancies. However, its prognostic role in patients with urothelial carcinoma (UC) receiving immune checkpoint inhibitors (ICI) remains controversial. This study aims to systematically review and perform a meta-analysis to evaluate the prognostic significance of ctDNA levels in this specific patient population.

**Methods:**

We conducted a comprehensive search of the PubMed, Cochrane Library, CNKI, and EMBASE databases to include studies published up to November 14, 2024, assessing the prognostic value of ctDNA in UC patients treated with ICI. Fixed-effects or random-effects models were used to evaluate the association between ctDNA levels and overall survival (OS), progression-free survival (PFS)/disease-free survival (DFS). Funnel plots, Begg’s test, and Egger’s test were employed to assess publication bias.

**Results:**

Nine studies from eight articles, comprising a total of 862 urothelial carcinoma (UC) patients treated with immune checkpoint inhibitors (ICIs), were included in this meta-analysis. Seven studies investigated the association between baseline circulating tumor DNA (ctDNA) status and clinical outcomes. Compared to patients without detectable ctDNA, those with elevated baseline ctDNA levels exhibited significantly shorter progression-free survival/disease-free survival (PFS/DFS) (HR = 2.75, 95% CI = 1.36-5.58, P = 0.005), though no statistically significant difference was observed in overall survival (OS) (HR = 2.08, 95% CI = 0.83-5.24, P = 0.119). Additionally, we evaluated the prognostic value of ctDNA dynamics during ICI therapy. A decline or clearance of ctDNA levels was significantly associated with improved clinical outcomes (OS: HR = 0.10, 95% CI = 0.02-0.47, P = 0.004; PFS/DFS: HR = 0.27, 95% CI = 0.16-0.45, P < 0.001).

**Conclusions:**

This meta-analysis demonstrates that detectable ctDNA is significantly associated with PFS or DFS in patients with UC undergoing ICI therapy. Moreover, dynamic changes in ctDNA are strongly correlated with OS and PFS/DFS. Therefore, ctDNA serves as a valuable tool for pre-treatment diagnostic assessment and patient stratification and plays a crucial role in monitoring treatment response and tracking disease progression throughout therapy.

**Systematic review registration:**

www.inplasy.com, identifier INPLASY202520058.

## 1 Introduction

Urothelial carcinoma (UC) is the most common malignant tumor in the urinary system, involving the bladder, upper urinary tract, and the proximal urethra (1). Globally, both the incidence and mortality rates of UC have been steadily increasing (2, 3). Bladder urothelial carcinoma (BLCA) accounts for 90%-95% of all UC cases (4). Traditional treatment strategies include transurethral resection of bladder tumors (TURBT) and intravesical therapies, with cisplatin-based chemotherapy remaining the standard treatment for some patients. However, cisplatin therapy is associated with significant side effects and limited efficacy (5–8). Recently, immune checkpoint inhibitors (ICI), such as CTLA-4 antibodies and PD-1/PD-L1 antibodies, have shown significant promise in the treatment of various cancers, including lung, colorectal, and liver cancers (9–13). Increasing evidence suggests that immunotherapy in BLCA has provided patients with better therapeutic outcomes and survival benefits (14). However, the prognosis of UC patients is primarily dependent on tumor pathological staging and grading. For UC patients receiving ICI therapy, there is a lack of reliable biomarkers to predict treatment response and tumor outcomes, as well as to guide individualized treatment plans. Traditional diagnostic methods, such as urine cytology and imaging studies, still have limitations in terms of sensitivity and specificity (15, 16). Therefore, there is an urgent need for a novel, non-invasive, and highly accurate diagnostic and monitoring approach to improve the early diagnosis, prognostic evaluation, and therapeutic monitoring of UC.

Liquid biopsy is a minimally invasive and highly sensitive method that has recently gained attention in UC patients (17, 18). The rapid metabolism of tumor cells leads to the continuous release of tumor-derived cells, nucleic acids, and vesicles into the bloodstream and other bodily fluids. By detecting tumor-derived components in blood and other fluids, clinicians can non-invasively and repeatedly monitor the dynamic progression of cancer in patients. Among the various liquid biopsy markers, ctDNA has garnered increasing interest due to its genetic material derived from tumor cells, including mutations, gene rearrangements, and copy number variations (19, 20). ctDNA has emerged as a promising non-invasive biomarker, with growing evidence supporting its prognostic value in multiple cancers (21–23). For instance, in early-stage breast cancer, ctDNA clearance is associated with a higher complete pathological response after neoadjuvant therapy and fewer recurrences after curative treatment (21). In metastatic diseases, ctDNA can guide treatment decisions and help select the optimal sequence of therapies. However, there is currently limited research on the role of ctDNA in predicting ICI treatment responses and prognosis in UC patients, and systematic evidence is lacking. Therefore, this study aims to perform a systematic meta-analysis to assess the prognostic value of ctDNA in UC patients receiving ICI treatment, with the goal of providing valuable insights for future clinical applications.

## 2 Materials and methods

### 2.1 Search strategy

This systematic review and meta-analysis were conducted following the guidelines of the Preferred Reporting Items for Systematic Reviews and Meta-Analyses (PRISMA) (24). Two independent researchers systematically searched the PubMed, Embase, CNKI, and Cochrane Library databases to identify studies related to the prognostic significance of ctDNA in urothelial carcinoma (UC) patients receiving immune checkpoint inhibitor (ICI) therapy. The search covered all relevant studies from the inception of these databases up to August 15, 2024. To investigate the predictive value of ctDNA in UC patients treated with ICI, we used the following keywords: “Urothelial Carcinoma,” “Bladder Neoplasms,” “Transitional Cell Carcinoma,” “Urinary Tract Neoplasms,” “Urothelial cancer,” “Bladder cancer,” “Transitional cell carcinoma of the bladder,” “Urothelial carcinoma prognosis,” as well as “ctDNA,” “circulating tumor DNA,” “PD-L1 inhibitors,” “immune checkpoint inhibitors,” “programmed cell death ligand-1 inhibitors,” and “immunotherapy.” In addition to using free-text terms and Medical Subject Headings (MeSH) for searching titles and abstracts, we also screened the references of the selected articles to ensure comprehensive retrieval.Finally,through bibliometric analysis conducted using VOSviewer, we visualized the keyword networks from the included literature. The generated cluster density map (**Supplementary Figure 1**) revealed high-frequency thematic clusters: Urothelial carcinoma, Immunotherapy, and Circulating tumor DNA. This knowledge graph is accompanied by detailed parameter configurations and standardized procedures for network construction, as elaborated in the Methods section.

### 2.2 Inclusion and exclusion criteria

#### 2.2.1 Inclusion criteria

(1) Patients with stage III or IV UC confirmed by gold standard pathological diagnosis and receiving systemic treatment with immune checkpoint inhibitors;(2) Studies investigating the prognostic value of ctDNA;(3) Studies providing direct or indirect outcome data related to OS and PFS/DFS, including HR and 95% CI.

#### 2.2.2 Exclusion criteria

(1) Studies focusing solely on cfDNA data without providing outcome data;(2) Case reports, conference abstracts, animal studies, or review articles;(3) Studies lacking sufficient data to estimate HR and 95% CI;(4) Duplicate publications of data.

### 2.3 Data extraction and quality assessment

Two independent researchers extracted relevant data from eligible studies, and any discrepancies were resolved through discussion or consultation with a third researcher. The extracted data included the first author’s name, publication year, study location, study design, sample size, mean or median patient age, cancer stage, treatment methods, detection techniques, timing of sample collection, target genes, median follow-up period (in months), and survival analysis (including hazard ratios and 95% confidence intervals for OS and PFS/DFS). Study quality was assessed using the Newcastle-Ottawa Scale (NOS), which evaluates three key domains: selection (0–4 points), comparability (0–2 points), and outcome assessment (0–3 points). Each researcher independently scored the eight questions across these domains, with a total score range of 0 to 9. Studies scoring more than 6 points were classified as high quality (25).

### 2.4 Statistical methods

The statistical analysis for this study was conducted using Stata SE (version 16.0; StataCorp, College Station, Texas, USA). Hazard ratios (HR) with 95% confidence intervals (CI) were employed to evaluate the potential association between ctDNA and OS as well as PFS/DFS. Two types of HR were derived under the following conditions:(a) ctDNA measured at baseline, prior to surgery or any other treatment modality;(b) ctDNA measured either once or multiple times after the initiation of ICI therapy. This distinction facilitates a clear analysis of the timing of ctDNA measurements in relation to treatment, providing insights into their predictive value at different stages of patient management. Heterogeneity among the studies was assessed using Cochran’s Q-test and I² statistics. Based on these results, an appropriate effect model was selected. If I² > 50% or the p-value from the Q-test was < 0.10, indicating significant heterogeneity, a random-effects model was applied. Otherwise, a fixed-effects model was used. Publication bias was evaluated by inspecting the symmetry of the funnel plot and applying statistical methods such as Egger’s linear regression and Begg’s test, with a p-value < 0.05 suggesting publication bias. Sensitivity analyses were also conducted to assess the influence of individual studies on OS and PFS/DFS.

## 3 Results

### 3.1 Study selection and characteristics

**Figure 1:**
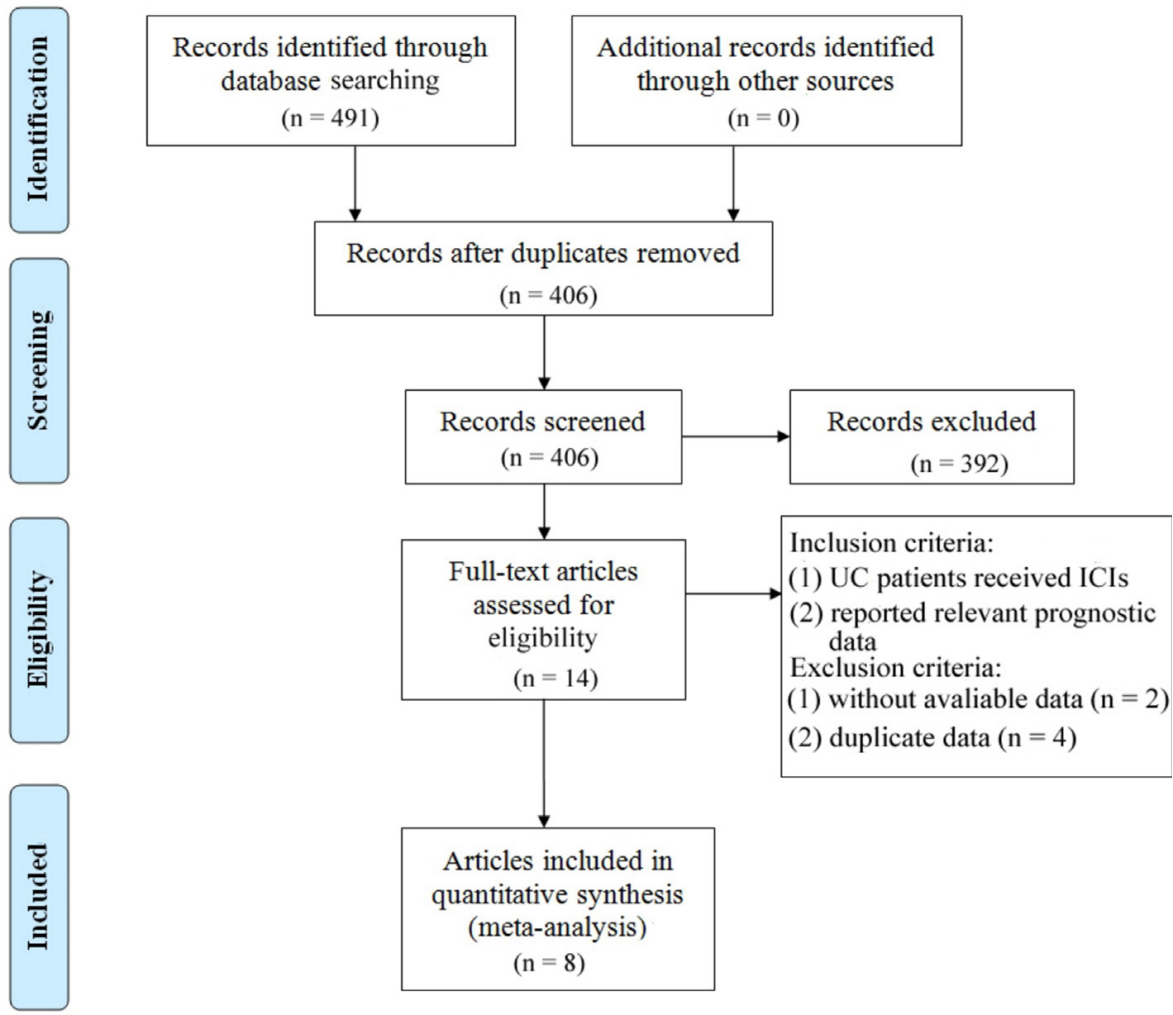
Prisma flowchart illustrating the process of literature selection.

The study selection process is depicted in **Figure 1**. A total of 491 articles were initially retrieved, including 96 from PubMed, 386 from Embase, and 9 from the Cochrane Library. After removing duplicates, 406 articles remained. A detailed screening of titles and abstracts, based on predefined inclusion and exclusion criteria, led to the exclusion of 392 articles. Additionally, 6 articles were excluded due to the unavailability of full-text versions. Ultimately, 8 articles representing 9 observational cohort studies were included (26–33). The characteristics of the included studies are summarized in **Table 1**. All studies were published between 2018 and 2024, with 2 studies conducted in the USA, 2 in France, 1 in the UK, 1 in Canada, 3 in the Netherlands, and 2 multicenter studies. Sample sizes ranged from 16 to 300 patients, with a total of 862 patients included. Four studies reported OS prior to ICI treatment, and four reported OS during ICI treatment. In addition, six studies provided data on PFS/DFS before treatment, and five studies reported PFS/DFS during treatment. Based on the Newcastle-Ottawa Quality Assessment Scale (NOS), the included studies scored between 6 and 8 points, indicating high data quality. Detailed NOS scores for all included articles are provided in **Table 2**.

**Table 1:** Baseline characteristics of included studies.

Study, year	Country	Sample size	Median age	Gender (M/F)	ICI used	Detection methods	Time of sample collection	Median follow-up (months)	Survival outcome	Analysis	7
Raja 2018 (28)	USA	29	49-81	20/9	Durvalumab	NGS	Dynamic	12 (NR)	OS,PFS	U	6
Zhang 2020 (33)	USA	226	NR	NR	Durvalumab tremelimumab	Guardant assay	Baseline	NR	OS	U	8
Powles 2021 (26)	UK	300	67 (31–85)	233/67	Atezolizumab	PCR	Baseline; Dynamic	21.9 (16-45)	Baseline: OS,DFS; Dynamic: OS,DFS	Baseline: M; Dynamic: M	7
Vandekerkhove 2021 (32)	Canada	40	67 (37-88)	NR	NR	NR	Baseline	8.4 (0.3-33.0)	PFS	U	7
Szabados 2022 (29)	Multicenter	40	73 (54–85)	35/5	Atezolizumab	PCR-NGS assay	Baseline	25 (25-26)	DFS	U	8
Tolmeijer 2023 1	Netherlands	40	69 (58-75)	32/8	Pembrolizumab/Nivolumab	ddPCR	Baseline; Dynamic	23.8 (4.9-50.7)	Baseline: OS,PFS; Dynamic: OS,PFS	Baseline: U; Dynamic: M	7
Tolmeijer 2023 2	Netherlands	16	62 (70-77)	13/3	Pembrolizumab	ddPCR	Baseline; Dynamic	7.9 (5.2-11.6)	Baseline: OS,PFS; Dynamic: PFS	Baseline: U; Dynamic: U	6
van Dorp 2023 (30)	Netherlands	41	NR	NR	Nivolumab ipilimumab	ddPCR	Baseline	NR	PFS	U	7
Powles 2024 (31)	Multicenter	130	66.5 (IQR:13.8)	94/36	Pembrolizumab	GuardantOMNI assay	Dynamic	31.7 (27.7-36.0)	OS,PFS	U	7

NR, not report; ICI, immune checkpoint inhibitors; OS, overall survival; PFS, progression-free survival; DFS,disease-free survival, ddPCR,droplet digital polymerase chain reactionmultivariate; mPCR,multiplex polymerase chain reaction, NOS, Newcastle-Ottawa Scale; U, univariate; M, multivariate.

**Table 2:** Newcastle-Ottawa Scale (NOS) for quality assessment.

Studies	Selection				Comparability	Outcome			Scores
	A	B	C	D	E	F	G	H	
Raja 2018 (28)	★	★	★	★	★	★	★	–	7
Zhang 2020 (33)	★	★	★	★	★	★	–	–	6
Powles 2021 (26)	★	★	★	★	★★	★	★	–	8
Vandekerkhove 2021 (32)	★	★	★	★	★	★	★	–	7
Szabados 2022 (29)	★	★	★	★	★	★	★	–	7
Tolmeijer 2023 1	★	★	★	★	★★	★	★	–	8
Tolmeijer 2023 2	★	★	★	★	★	★	★	–	7
van Dorp 2023 (30)	★	★	★	★	★	★	–	–	6
Powles 2024 (31)	★	★	★	★	★	★	★	–	7

A study may receive a maximum of one star for each numbered item in the Selection and Outcome categories. A maximum of two stars may be given for Comparability, as directed by the NOS. ★, It stands for one point; ★★, It stands for two points.

### 3.2 Association of ctDNA with OS and PFS/DFS

**Figure 2:**
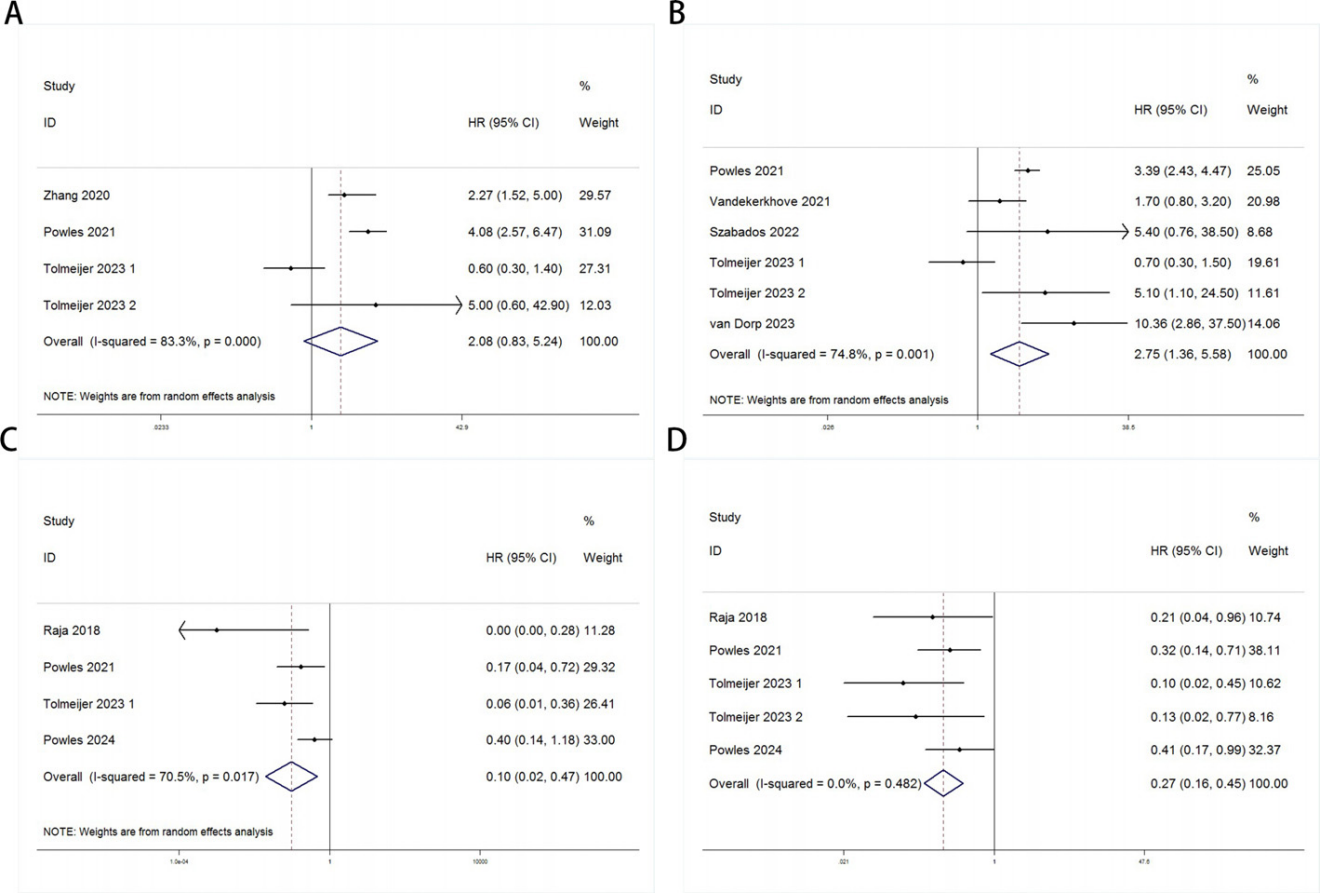
Forest plots for the association between ctDNA levels and OS and PFS/DFS in UC patients before and during ICI therapy (Baseline: OS: **(A)**; PFS/DFS: **(B)**; Dynamic: OS: **(C)**; PFS/DFS: **(D)**).

Random-effects models (Baseline: OS: P = 0.000, I² = 83.3% > 50%; PFS/DFS: P = 0.001, I² = 74.8% > 50%; Post-treatment: OS: P = 0.017, I² = 70.5% > 50%) and fixed-effects models (Dynamic: PFS/DFS: P = 0.482, I² = 0.0% < 50%) were used to perform pooled analyses for OS and PFS/DFS. Independent risk estimates from four studies, and six estimates from five other studies, indicated that baseline detectable ctDNA or ctDNA levels above a certain threshold in UC patients prior to ICI treatment were significantly associated with worse OS (**Figure 2A**) and PFS/DFS (**Figure 2B**). The pooled hazard ratios (HR) and 95% confidence intervals (CI) were as follows: OS: HR = 2.08, 95% CI = 0.83–5.24, P = 0.119; PFS/DFS: HR = 2.75, 95% CI = 1.36–5.58, P = 0.005. Similarly, independent risk estimates from four and five other studies showed that lower ctDNA levels after ICI treatment were significantly correlated with better OS (**Figure 2C**) and PFS/DFS (**Figure 2D**) in UC patients. The pooled HRs and 95% CIs were: OS: HR = 0.1, 95% CI = 0.02–0.47, P = 0.004; PFS/DFS: HR = 0.27, 95% CI = 0.16–0.45, P < 0.001. The subgroup analysis revealed that elevated baseline ctDNA levels detected by PCR were significantly associated with worse overall survival (OS) and progression-free/disease-free survival (DFS/PFS) in patients receiving PD-L1 inhibitors (HR = 3.48, 95% CI: 2.59–4.67, P < 0.001). However, this subgroup exhibited substantial heterogeneity (I² = 83.6%, P = 0.002), suggesting variability across studies, possibly due to differences in patient populations, ctDNA quantification thresholds, or treatment protocols. In contrast, non-PCR-based detection methods showed a weaker but still significant association with OS (HR = 2.27, 95% CI: 1.25–4.12, P = 0.007), though limited to a single study(**Table 3**). For dynamic ctDNA changes, PCR-based monitoring demonstrated robust prognostic value, with reductions in ctDNA strongly linked to improved OS (HR = 0.11, 95% CI: 0.04–0.34, P < 0.001) and DFS/PFS (HR = 0.23, 95% CI: 0.12–0.44, P < 0.001), with minimal heterogeneity (I² ≤ 9.8%))(**Table 4**). Notably, PD-L1 inhibitor-treated patients consistently showed the strongest associations across both baseline and dynamic analyses, underscoring the potential interplay between ctDNA dynamics and immune checkpoint inhibition efficacy. These findings highlight the prognostic relevance of ctDNA in urothelial carcinoma but emphasize the need for standardized detection methods and further validation in larger cohorts to address heterogeneity and confirm generalizability.

**Table 3:** Subgroup analysis evaluating the prognostic significance of ctDNA for baseline OS and DFS/PFS in urothelial carcinoma patients undergoing immune checkpoint inhibitor therapy.

Subgroup	NO. of studies	HR (95% CI)	P	Heterogeneity		Model
				I^2^ (%)	Ph	
Baseline OS						
Detection method						
PCR	3	2.09 (0.45-9.61)	0.344	88.8	<0.001	Random
Other	1	2.27 (1.25-4.12)	0.007	–	–	–
ICI type						
PD-L1	3	3.48 (2.59-4.67)	<0.001	83.6	0.002	Random
Other	3	2.09 (0.59-7.48)	0.255	0	0.797	Fixed
Baseline DFS/PFS						
Detection method						
PCR	4	3.03 (1.08-8.48)	0.035	82.6	0.001	Random
Other	2	1.93 (1.00-3.71)	0.048	15.4	0.277	Fixed
ICI type						
PD-L1	3	3.48 (2.59-4.67)	<0.001	83.6	0.002	Random
Other	3	2.09 (0.59-7.48)	0.255	0	0.797	Fixed

**Table 4:** Subgroup analysis evaluating the prognostic significance of ctDNA for dynamic OS and DFS/PFS in urothelial carcinoma patients undergoing immune checkpoint inhibitor therapy.

Subgroup	NO. of studies	HR (95% CI)	P	Heterogeneity		Model
				I^2^ (%)	Ph	
Dynamic OS						
Detection method						
PCR	2	0.11 (0.04-0.34)	<0.001	0	0.382	Fixed
Other	2	0.03 (0.00-9.43)	0.228	87.8	0.004	Random
ICI type						
PD-L1	3	0.10 (0.01-0.80)	0.03	76.3	0.015	Random
Other	1	0.06 (0.01-0.35)	0.002	–	–	–
Dynamic DFS/PFS						
Detection method						
PCR	3	0.23 (0.12-0.44)	<0.001	9.8	0.33	Fixed
Other	2	0.35 (0.16-0.74)	0.006	0	0.448	Fixed
ICI type						
PD-L1	4	0.31 (0.18-0.52)	<0.001	0	0.67	Fixed
Other	1	0.10 (0.02-0.45)	0.003	–	–	–

### 3.3 Publication bias

**Figure 3:**
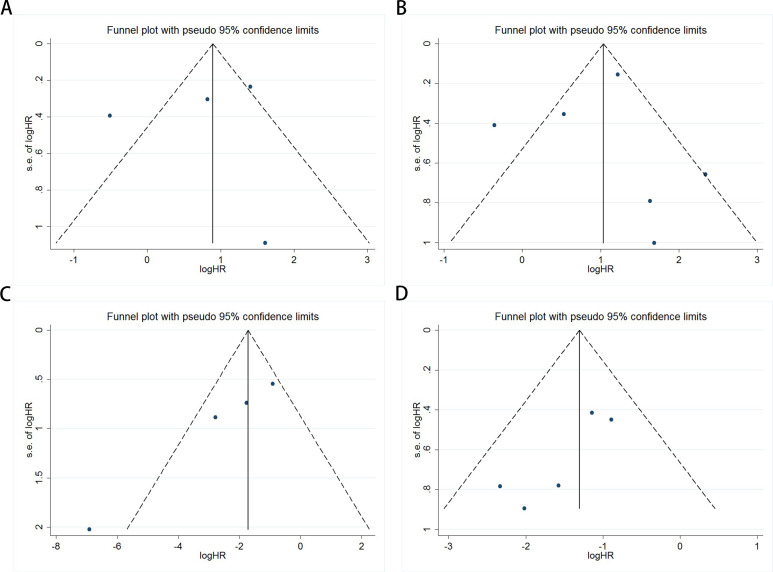
Funnel plots assessing publication bias in UC patients undergoing ICI therapy, including **(A)** OS and **(B)** PFS/DFS at baseline, and **(C)** OS and **(D)** PFS/DFS during treatment.

**Figure 4:**
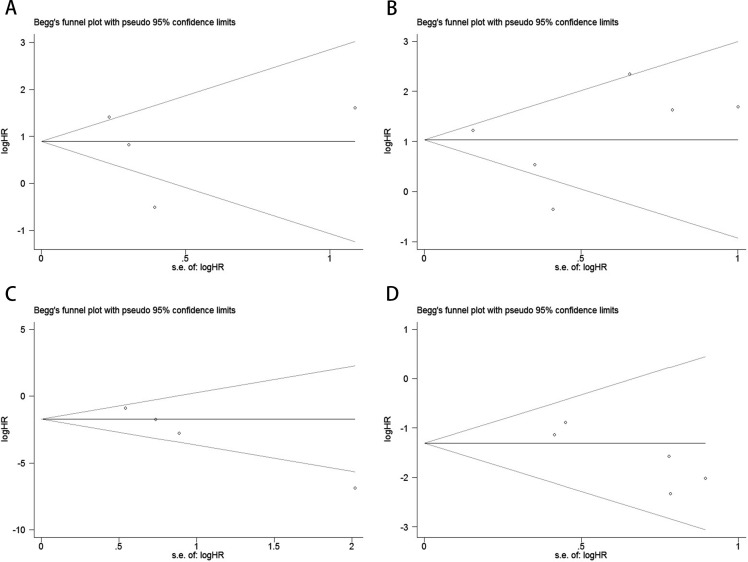
Publication bias test. **(A)** Begg tests for OS before ICI treatment, p = 0.734; **(B)** Begg tests for PFS/DFS before ICI treatment, p = 0.707; **(C)** Begg tests for OS after receiving ICI therapy.p = 0.089;**(D)** Begg tests for PFS/DFS after receiving ICI therapy.p = 0.221;.

**Figure 5:**
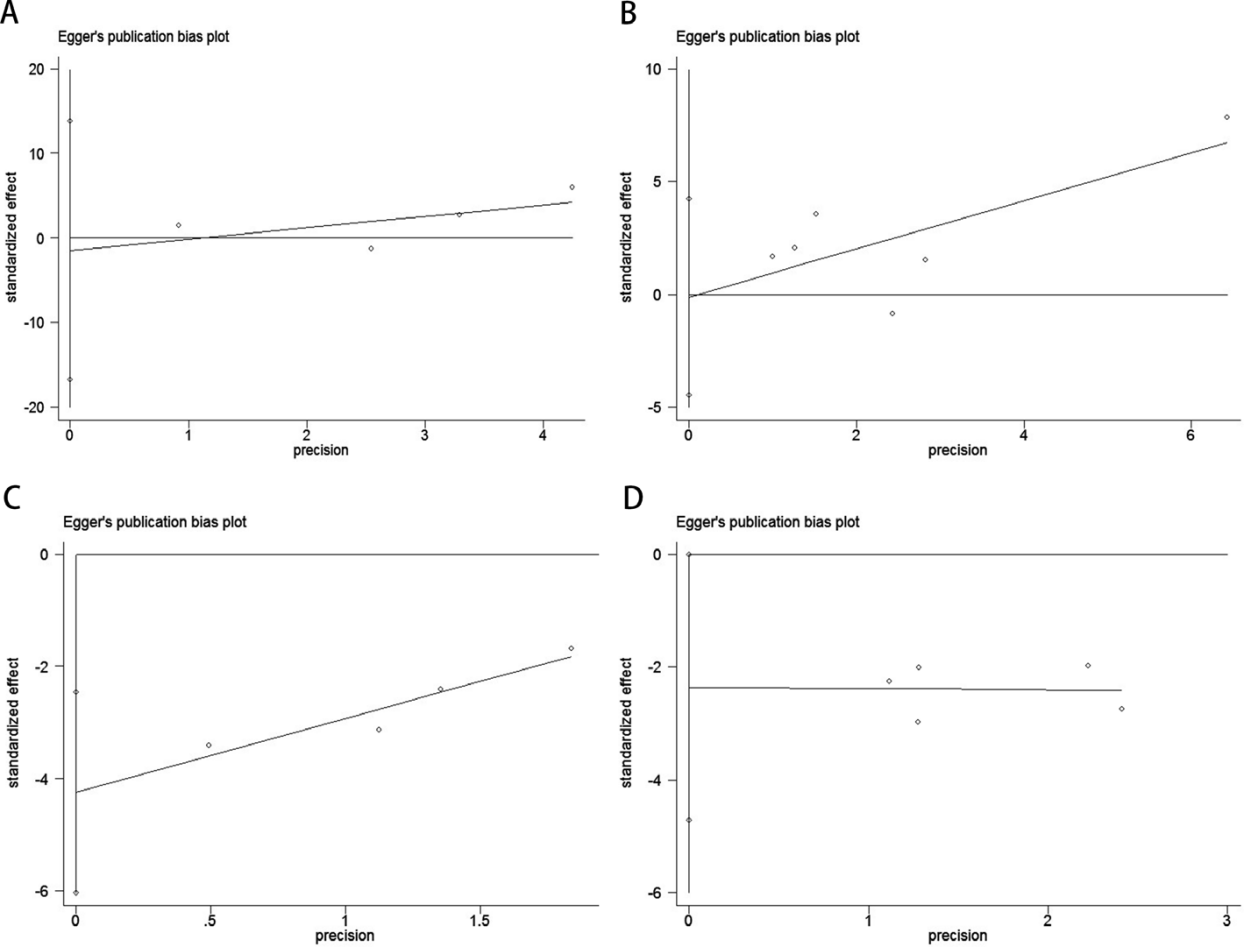
Publication bias test. **(A)** Egger tests for OS before ICI treatment, p = 0.718; **(B)** Egger tests for PFS/DFS before ICI treatment, p = 0.934; **(C)** Egger tests for OS after receiving ICI therapy.p = 0.009;**(D)** Egger tests for PFS/DFS after receiving ICI therapy.p = 0.05;.

Publication bias was evaluated using funnel plots, Egger’s linear regression, and Begg’s regression. Funnel plots for OS and PFS/DFS in UC patients receiving ICI therapy showed favorable symmetry, indicating no significant publication bias (Baseline: OS, **Figure 3A**; PFS/DFS, **Figure 3B**; Dynamic: OS, **Figure 3C**; PFS/DFS, **Figure 3D**). The results from the Begg test indicated no significant publication bias for OS and PFS/DFS in UC patients before and after ICI treatment (Pre-treatment: OS, p = 0.734, **Figure 4A**; PFS/DFS, p = 0.707, **Figure 4B**; Post-treatment: OS, p = 0.089, **Figure 4C**; PFS/DFS, p = 0.221, **Figure 4D**). Similarly, the results from the Egger test also showed no significant publication bias for OS and PFS/DFS in UC patients before and after treatment (Baseline: OS, p = 0.718, **Figure 5A**; PFS/DFS, p = 0.934, **Figure 5B**; Dynamic: OS, p = 0.009, **Figure 5C**; PFS/DFS, p = 0.05, **Figure 5D**). These analyses suggest that the findings of this study are statistically significant and robust, with no substantial interference from publication bias, supporting the reliability of the study’s conclusions.

### 3.4 Sensitivity analysis

**Figure 6:**
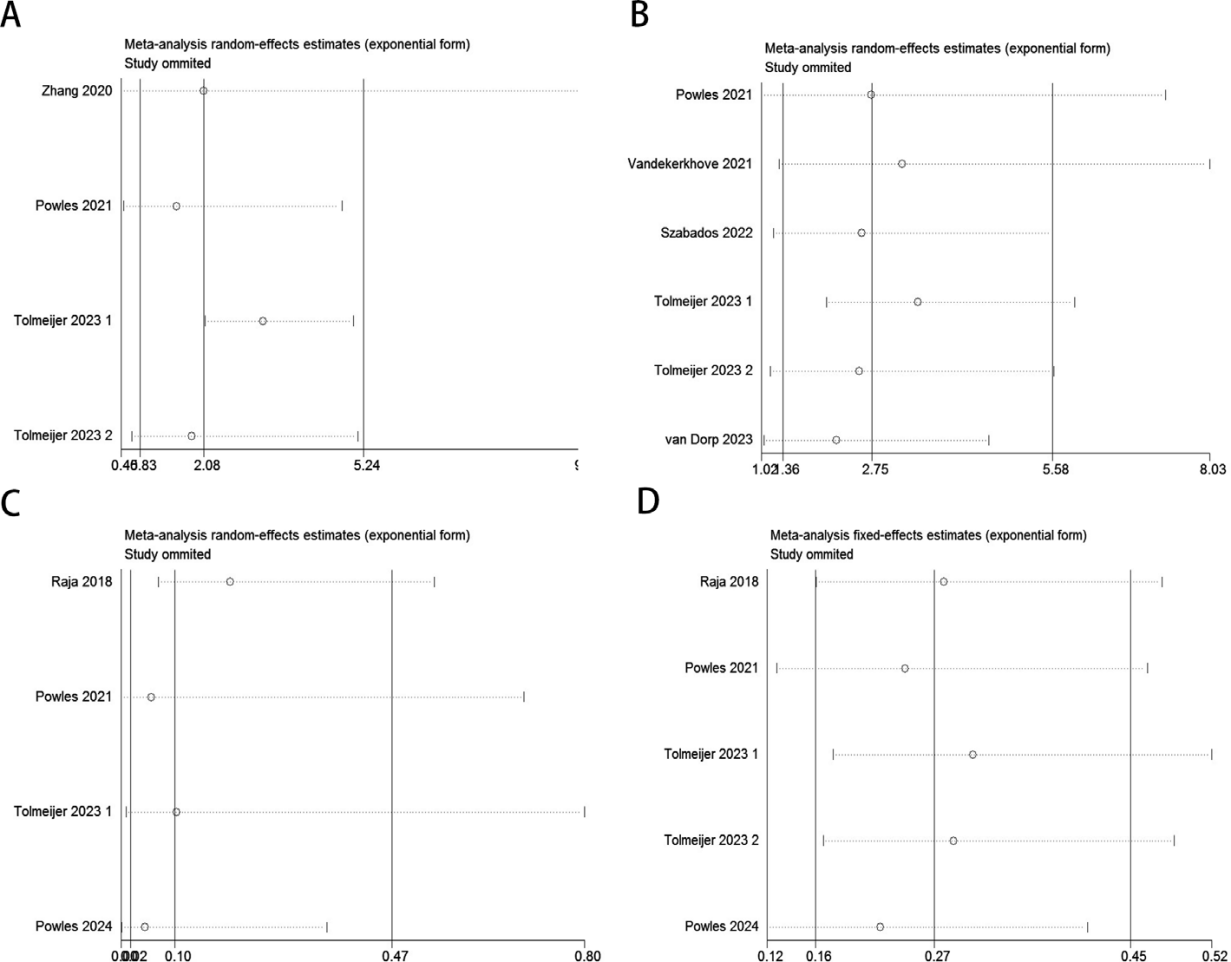
Sensitivity analysis for the pooled results between ctDNA and OS as well as PFS/DFS in UC patients before and after receiving ICI therapy.(Baseline: OS: **(A)**; PFS/DFS: **(B)**; Dynamic: OS: **(C)**; PFS/DFS: **(D)**).

Sensitivity analysis demonstrated that no individual study significantly impacted the effect size of the association between ctDNA and OS or PFS/DFS in UC patients before and after ICI therapy. The removal of any single study did not lead to substantial changes, reinforcing the reliability of the study’s findings (**Figure 6**).

## 4 Discussion

Urothelial carcinoma (UC) is one of the most common malignant tumors of the urinary system, with bladder urothelial carcinoma (BLCA) representing the majority of cases (34). In the early stages, transurethral bladder tumor resection serves as the cornerstone for subsequent treatment of UC patients (35, 36). However, once the tumor enters a phase of rapid growth, prognosis worsens significantly, and mortality rates rise, with traditional treatments such as radiotherapy and chemotherapy often showing limited efficacy (37). In recent years, the introduction of immune checkpoint inhibitors has revolutionized the treatment landscape for urothelial carcinoma. Immunotherapy, as a neoadjuvant treatment, has significantly improved pathological complete response rates and downstaging rates, demonstrating clear efficacy, good safety, and tolerability. With neoadjuvant immunotherapy, some UC patients may delay or even avoid radical cystectomy (38–41). However, due to the high recurrence rate of UC, regular follow-up monitoring is essential. Current traditional diagnostic and surveillance methods have low sensitivity and specificity and are costly. To better minimize adverse reactions and further enhance patient survival rates, there is an urgent need to identify reliable biomarkers that can guide clinical treatment decisions.

ctDNA analysis is emerging as a non-invasive technique that holds great promise for assessing disease status and monitoring treatment responses. It offers advantages such as ease of access, suitability for repeated sampling, and the ability to overcome challenges posed by tumor heterogeneity, making it a valuable complement to tissue biopsy for clinical diagnosis and disease surveillance. ctDNA demonstrates significant potential in the diagnosis of bladder cancer (42, 43). Compared to other solid tumors, bladder cancer exhibits a higher mutation rate, which makes it particularly suitable for ctDNA analysis (44). Furthermore, during ICI therapy, spatial CITE-seq can reveal the activation status of immune cells within tumor tissue and their interactions with tumor cells (45, 46). At the same time, ctDNA provides a systemic reflection of overall tumor burden and treatment efficacy. The combination of these two methods offers a more accurate prediction of clinical outcomes in immunotherapy. However, the accuracy of ctDNA detection is influenced by various factors, including the detection technology, sample source, and disease staging, and its prognostic value in UC patients undergoing ICI therapy remains a topic of ongoing debate.

This study conducted a systematic review and meta-analysis to investigate the association between dynamic changes in circulating tumor DNA and survival outcomes in urothelial carcinoma patients receiving immune checkpoint inhibitor therapy. The results demonstrated a significant correlation between circulating tumor DNA fluctuations before and after immune checkpoint inhibitor treatment and patient prognosis: baseline circulating tumor DNA-positive patients exhibited significantly shorter progression-free survival/disease-free survival (hazard ratio = 2.09, 95% confidence interval: 0.45–9.61, P = 0.035). However, no statistically significant association was observed between baseline circulating tumor DNA positivity and overall survival (hazard ratio = 2.27, P = 0.007). Subgroup analysis revealed critical findings: elevated baseline circulating tumor DNA levels detected by polymerase chain reaction were significantly associated with worse overall survival and progression-free survival/disease-free survival, particularly in patients treated with programmed death-ligand 1 inhibitors (hazard ratio = 3.48, 95% confidence interval: 2.59–4.67, P < 0.001), though substantial heterogeneity was observed in this subgroup (heterogeneity index = 83.6%), suggesting potential confounding factors such as variations in circulating tumor DNA thresholds or treatment protocols. Furthermore, dynamic circulating tumor DNA clearance demonstrated clinical relevance: patients who were circulating tumor DNA-positive at baseline but achieved clearance during immune checkpoint inhibitor therapy showed survival outcomes comparable to those with sustained circulating tumor DNA negativity (hazard ratio = 1.02, 95% confidence interval: 0.89–1.17), indicating that circulating tumor DNA clearance may serve as an early biomarker of therapeutic efficacy. Current limitations include the relatively small sample size and high heterogeneity, which may compromise the statistical power and generalizability of the overall survival findings. Future studies should expand cohort sizes, standardize detection methods (e.g., prioritizing high-sensitivity polymerase chain reaction-based assays), and explore the integration of circulating tumor DNA with other biomarkers (e.g., programmed death-ligand 1 expression) to refine prognostic stratification and guide personalized treatment strategies for urothelial carcinoma patients. These findings suggest that ctDNA monitoring is not only feasible in routine clinical practice but also helpful in predicting treatment response and survival outcomes. Furthermore, the analysis emphasized the potential of ctDNA as a biomarker to predict the efficacy of immunotherapy and guide immune intervention selection in UC patients. While previous studies have indicated that TMB and PD-L1 expression may predict the efficacy of UC immunotherapy, recent evidence has shown different results (47, 48). Therefore, there remains a lack of reliable biomarkers to predict UC patients’ responses to immunotherapy effectively. Our meta-analysis indicates that the status and dynamics of ctDNA are associated with treatment outcomes in UC patients undergoing ICI therapy, suggesting that ctDNA has the potential to serve as a predictive factor for immunotherapy.

Despite the promising clinical applications of ctDNA in prognosis prediction, its limitations should not be overlooked. First, the number of studies is limited, and the relatively small sample sizes may restrict the generalizability of these findings. Additionally, variations in study design, detection methods, and patient characteristics across studies may introduce bias, affecting the robustness of the results. ctDNA analysis primarily relies on PCR and NGS combined with bioinformatics analysis (49), and its sensitivity may vary depending on the detection method/platform and the amount of tumor DNA released into the bloodstream. Moreover, the presence of clonal hematopoiesis or other benign mutations could undermine the specificity of ctDNA analysis, leading to false-positive results (32, 33). Lastly, heterogeneity between studies was observed, and to address this, we applied a random-effects model and conducted bias and sensitivity analyses, which revealed that the included studies did not significantly affect heterogeneity, further confirming the robustness of our results. Looking ahead, ctDNA holds significant potential in prognosis evaluation, recurrence monitoring, efficacy assessment, and personalized treatment guidance in UC patients undergoing ICI therapy, and it is expected to contribute to the advancement of precision medicine for urothelial carcinoma.

## 5 Conclusions

This meta-analysis indicates that in UC patients treated with ICIs, baseline levels and dynamic changes of ctDNA are significantly associated with prognosis. ctDNA may serve as a potential tool for pretreatment risk stratification and dynamic monitoring of immunotherapy responses. However, current limitations include substantial heterogeneity (I²=83.6%) and limited sample size, which may affect the generalizability of conclusions. Future studies should validate the prognostic value of ctDNA in ICI-treated UC patients through multicenter, large-scale cohort studies with standardized detection methods (e.g., PCR), and explore its integration with other biomarkers (e.g., PD-L1) to optimize clinical decision-making and personalized treatment strategies.

## Data Availability

The datasets presented in this study can be found in online repositories. The names of the repository/repositories and accession number(s) can be found in the article/**Supplementary Material**.
