# Supplementary figures and images for: Prognostic significance of circulating tumor DNA in urothelial carcinoma patients undergoing immune checkpoint inhibitor therapy: a systematic review and meta-analysis
Source: Front Immunol. 2025 Apr 29;16:1574449. doi: 10.3389/fimmu.2025.1574449 (PMC12069302; doi:10.3389/fimmu.2025.1574449)

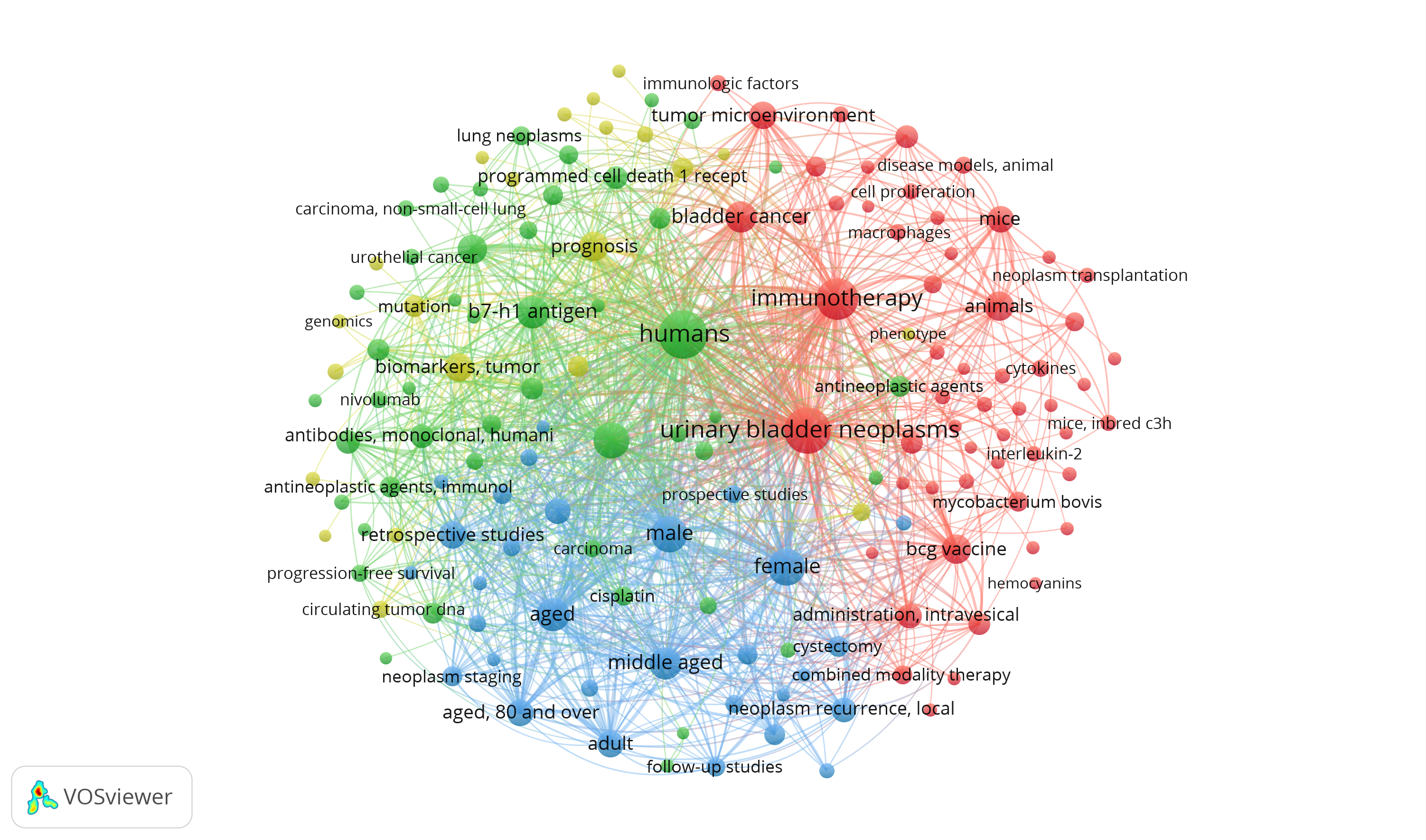

Supplement: Supplementary file 1 [file Image1.png]
